# Factors Associated with Cervical Cancer Screening Uptake: Implications for the Health of Women in Jordan

**DOI:** 10.1155/2020/9690473

**Published:** 2020-03-21

**Authors:** Suzanne Q. Al-amro, Muntaha K. Gharaibeh, Arwa I. Oweis

**Affiliations:** ^1^USAID Health Service Delivery, Amman Wadi Saqra, P.O. Box 851275, 11185, Jordan; ^2^Jordan University of Science & Technology/Irbid, P.O. Box 3030, 22110, Jordan; ^3^WHO, Regional Office, Nasr City, Cairo, Egypt

## Abstract

**Purpose:**

The existing factors that influence cervical cancer screening uptake worldwide do not necessarily reflect the situation in Jordan. Therefore, the aim of this study was to determine the factors associated with cervical cancer screening uptake among Jordanian women.

**Methods:**

In this cross-sectional study, 500 married Jordanian women aged 21 to 65 years were recruited from eight nongovernmental organisations and community settings in Amman. Data were collected with a self-administered questionnaire regarding sociodemographic and reproductive data, a health utilisation data form, and scales on the perceived benefits of screening, perceived barriers to screening, perceived susceptibility to cervical cancer, and perceptions regarding the severity of cervical cancer. Descriptive statistics, multivariate logistic regressions, and independent *t*-tests were used in the data analysis.

**Results:**

Among the 500 age-eligible women, only 156 (31.2%) had been screened for cervical cancer. Healthcare provider encouragement, years of marriage (odds ratio (OR) = 5.24, confidence interval (CI) = 95%, *p* = 0.00), and use of the private healthcare sector (OR = 2.20, CI = 95%, *p* = 0.012) were significant predictors of cervical cancer screening.

**Conclusion:**

Cervical cancer screening uptake among Jordanian women is significantly low; determining factors for the decision to undergo screening include encouragement from the healthcare provider, the number of years of marriage, and use of the private healthcare sector. To improve uptake, structured screening programmes need to be implemented in collaboration with national partners and institutions to decrease the incidence of cervical cancer in Jordan.

## 1. Background

Cancer of the cervix uteri is one of the most common cancers in developing countries and the third most common cancer among women worldwide, with an estimated 569,847 new cases and 311,365 deaths in 2018 as reported by Bruni et al. [1]. Overall, the mortality rates in developing countries are about four times higher than those in industrialised countries, with 80–85% of cervical cancer deaths occurring in developing countries according to Mupepi et al. [2] and Ferly et al. [3]. In 2010, cervical cancer killed 200,000 women globally [4].

Jordan is a Mediterranean high- to middle-income country with a total population of 10,101,994, of which, 48.5% are female. The percentage distribution of married Jordanian females aged 15 years and above is 56.6% (Department of Statistics (DoS), 2019) [5]. Recent estimates indicate that cervical cancer is the 7th leading cause of death in Jordanian women aged 15-44 years, and 61 die from the disease [6].

Early screening to detect cases at the precancerous stage when it requires simple management can prevent cervical cancer. The most internationally accepted and cost-effective method of screening is the Pap test as reported by Hawkins et al. [7].

Cervical cancer usually develops slowly, which means that most cases can be identified and managed when screening is performed regularly [8–10]. The Pap test has been shown to save women from unnecessary mortality and morbidity. For instance, in the case of Delhi, India, it has been estimated that the mortality rate from cervical cancer has decreased by 50–70% since the introduction of population-based screening [11].

A review of guidelines and cervical cancer screening recommendations indicate that screening should begin a few years after the initiation of sexual activity. A woman does not need to be screened after a total hysterectomy unless the reason for the surgery was cervical cancer [12].

In Jordan, the incidence rate of cervical cancer screening is still low and unsatisfactory. Furthermore, screening is not undertaken by the majority of Jordanian women compared with women in other countries in the same geographic region as reported by Al Nsour et al. [13]. This low incidence is influenced by many factors, including sociodemographic characteristics, health service utilisation, perceived benefits of screening, perceived barriers to screening, perceived susceptibility to cervical cancer, and perceived seriousness regarding the severity of cervical cancer.

The sociodemographic factors associated with cervical cancer screening uptake are age, marital status, education level, employment status, and income level. These factors influence women's screening practices and have been investigated by many researchers interested in the promotion of cancer screening [14, 15].

Recently, a nationally representative sample of Jordanian women was analysed to examine the association between cervical cancer screening practices and sociodemographic factors. Results indicated that the highest incidence of cervical cancer screening (35.8%) exists among women aged 35–44 years, whereas the lowest (11.0%) is found among women aged 65 years and above. Moreover, the incidence of cervical cancer screening is greater among women with high levels of education, employed women, and women with high income levels [13].

The data on cervical cancer and the Pap smear test in older women and women with low levels of education in Jordan are insufficient [16]. Furthermore, the incidence rates of cervical cancer screening among a nationally representative sample of the married women in 14,564 households in Jordan are 2.8% for women aged 15–19 years, 9.2% for women aged 20–29 years, 19.7% for women aged 30–39 years, and 25.7% for women aged 40–49 years (WHO/ICO Information Centre on HPV and Cervical Cancer, 2010) [17].

Certain other factors influence women's cancer screening practices, such as health service utilisation (which consists of health insurance, preference for a female healthcare provider, physician recommendation, and healthcare provider encouragement) and the quality, availability, and accessibility of healthcare services [18].

In Jordan, the Ministry of Health (MoH) provides health insurance for 80% of the population and promotes preventive measures, such as cervical cancer screening, via the public sector healthcare organisation (Department of Statistics (DoS) and ICF International, 2013) [19]. Many Jordanian women who have had a Pap smear prefer a female doctor or female nurse; specifically, approximately 62.8% have expressed a preference for a female physician [20]. Additionally, approximately 86.2% of Jordanian women have had at least one Pap smear because doing so was recommended by a healthcare provider or physician [16].

Cervical cancer screening uptake is also associated with perceived benefits of screening, perceived barriers to screening, susceptibility to cervical cancer, and seriousness regarding the severity of cervical cancer, all of which motivate an individual to engage in cancer screening practices. The perceived benefits of screening refer to women's perceptions that cervical cancer screening will lead to early detection and treatment as reported by Parmer and Taylor [18].

Leyva et al. found that women supported the statement, “Screening is important to perform.” They acknowledged that being screened regularly for cervical cancer by means of a Pap test gave them peace of mind and identified a problem before it could become a major health issue [21].

Perceived barriers to screening are also shown to influence screening practices. The most reported barriers are fear of cancer screening (fear of negative results, the procedure, the instrument, the expectation of pain, or bleeding), embarrassment, a fatalistic belief regarding screening, lack of time, husband's disapproval, attitude of healthcare provider, and the absence of female physicians [22, 23].

Perceived susceptibility to cervical cancer is another factor that influences women's attitudes towards screening. Ibekwe found that perceived susceptibility significantly influences cervical cancer screening; women who perceived susceptibility to a high degree were 3.2 times more likely to undergo screening for cervical cancer than those with low levels of perceived susceptibility [24].

Perceived seriousness regarding the severity of cervical cancer also influences a woman's decision to be screened. Guilfoyle et al. found that older women tended to be fatalistic concerning their decision to be screened for cervical cancer, believing either that screening had no benefits or that their lives were in the hands of God [25].

However, the factors that have been identified as influential in cervical cancer screening uptake worldwide do not necessarily reflect the situation in Jordan. Therefore, investigating the factors associated with cervical cancer screening uptake within the context of Jordanian women's culture, religion, and economic status will provide a clearer picture about the significant predictors that influence the decision to submit to the cervical Pap smear procedure.

## 2. Methods

### 2.1. Design

A cross-sectional/correlation design was utilised to facilitate the gathering of data from the subset of a population and identify independent variables and associations among them [26].

### 2.2. Setting and Participants

The study was conducted in eight nongovernmental organisations (NGOs) located in the city of Amman, the capital of Jordan, to ensure an accurate representation of the sociodemographic distribution. Data were collected between October and December 2013.

The target population included married Jordanian women aged 21 to 65 years old who resided in different parts of Amman. Since the study included Muslim women, unmarried women and adolescents were not included because they are not sexually active; premarital sex is prohibited in Islam. Women who had had hysterectomies were excluded from the study. A nonprobability convenience sample of 500 women was recruited. Convenience sampling helps with the recruitment of a heterogeneous sample with considerable variations in age, educational level, experience, perceptions, and attitudes [26, 27].

### 2.3. Data Collection Procedure

The researcher explained the purposes and significance of the study to the directors of the participating NGOs to gain their endorsement of the data collection procedure. The purposes and significance of the study were explained to each potential participant who was invited to take part in the study. For illiterate women, a structured interview was conducted using the same questionnaire. The questionnaire was numerically coded before being administered to the women.

### 2.4. Measurement Instrument

A self-administered three-part questionnaire was used to collect data; it consisted of sociodemographic and reproductive data information, a health service utilisation data form, and perceived benefits, barriers, susceptibility, and seriousness regarding severity scales.

### 2.5. Sociodemographic and Reproductive Data Information

The first section of the questionnaire consisted of six questions aimed at obtaining sociodemographic data on participating women name, age, marital status, educational level, working status, type of employment, family's monthly income, and 14 questions pertaining to reproductive data, as well as questions regarding women's age at marriage, age at first pregnancy, number of pregnancies and living children, miscarriage history, years of marriage, duration of oral contraceptive use, smoking status, family history of cervical cancer, and history of sexually transmitted diseases.

### 2.6. Health Service Utilisation Data Form

The second section consisted of 14 questions about the availability of health coverage, health insurance type, availability and accessibility of healthcare services, preference for a female healthcare provider, physician recommendation, and encouragement provided by the healthcare provider.

### 2.7. Perceived Benefits, Barriers, Susceptibility, and Seriousness Scales

The third section used a four-point Likert scale ranging from 4 (strongly agree) to 1 (strongly disagree) to assess women's perceptions about cervical cancer and screening. The four scales were adapted from a study on cervical cancer screening beliefs among young Hispanic women undertaken by Byrd et al. [28].

The perceived benefits of cervical cancer screening scale measured women's perceptions that cervical cancer screening would result in early detection of cervical cancer, improve the chances of infertile women becoming pregnant, and decrease the chance of having an abortion. The scale consisted of five items. The total score of perceived benefits ranged from 5 to 20. The highest score represented highly perceived benefits of screening, and the lowest score represented minimally perceived benefits.

The perceived barriers to cervical cancer screening scale measured women's perceptions of the obstacles preventing them from being screened for cervical cancer. The scale consisted of 10 items. The total score for perceived barriers ranged from 10 to 40. The highest score represented highly perceived barriers to screening, and the lowest score represented minimally perceived barriers.

The perceived susceptibility to cervical cancer scale measured participants' perceptions that every woman of childbearing age and all older women were more susceptible to cervical cancer and that an increased number of pregnancies increased one's susceptibility to cervical cancer. The scale consisted of four items. The total score for perceived susceptibility to cervical cancer ranged from 4 to 16. The highest score represented high levels of perceived susceptibility to cervical cancer, and the lowest score represented low levels of perceived susceptibility.

The perceived seriousness regarding the severity of cervical cancer scale measured a woman's sense of seriousness regarding the severity of cervical cancer, possibilities for treatment, and other effects, including infertility. The scale consisted of six items, and the total score ranged from 6 to 24. The highest score represented a high level of perceived seriousness regarding the severity of cervical cancer, and the lowest score represented a low level of perceived seriousness.

### 2.8. Validity and Reliability of Measures

The questionnaire was initially developed in English and subsequently translated into Arabic, then back translated into English by a panel of four bilingual Ph.D. experts based in Jordanian universities who work in the field of maternal health.

These experts were asked to rate the relevancy and representativeness of each item in relation to the study topics. The original survey instrument consisted of 29 items. However, on the advice of the panel, four items were deleted. In addition, some phrases were reworded and the number of responses was reduced from five to four. Further, the five-point Likert-type scale was replaced with a four-point one.

The psychometric properties of the modified Arabic language survey instrument were evaluated in a pilot study involving 50 women to ensure cultural acceptance, ascertain the time needed to complete the questionnaire, and test the reliability of the measures. The women who took the pilot test found the instrument easy to understand and needed only 15 to 20 minutes to complete it. Cronbach's alphas for the pilot study and main study were 0.75 and 0.78, respectively, indicating a good level of reliability.

### 2.9. Procedure and Ethical Consideration

The approval of the Institutional Review Board of the Jordan University of Science and Technology was sought before conducting the study. Permission to conduct the study was also requested and obtained from the directors of the selected NGOs. Upon agreeing to the terms of the study, each participant was asked to sign a consent form that clearly identified the nature of her participation and her right to confidentiality and anonymity, as well as the right to withdraw from the study at any time.

### 2.10. Data Analysis

The Statistical Package for the Social Sciences (SPSS) version 17 for Windows was used for the data analysis. For all statistical analyses, the level of significance was set at 0.05. Both descriptive and inferential analyses were used. Prior to the analysis, data were cleaned by detecting errors, missing data, and outliers from data entered into SPSS for the descriptive statistics analysis.

## 3. Results

### 3.1. Sociodemographic and Gynaecological Characteristics of Participants

Table 1 shows the frequency and percentage distribution of the sociodemographic and reproductive characteristics of participants. The mean age of the study participants was 38.64 ± 9.39 years. The average family income per month was 800.20 ± 551 Jordanian dinar (JD), with a range of 170–5,000 JD. The majority of participants were married (91.80%). Approximately 66.00% had high levels of education (diploma or university degree), and 61.20% were employed. Approximately 59.20% of the employed participants worked for a governmental organisation, and 37.30% worked in the private sector.

The participants' mean age at marriage was 22.48 ± 4.13 years and ranged from 13 to 42 years. The mean age at first pregnancy was 23.22 ± 4.04 years and ranged from 15 to 43 years. The mean for years of marriage was 15.53 ± 10.07, and the mean number of pregnancies was 4.36 ± 2.27, ranging from 1 to 13 pregnancies. The mean number of living children was 3.68 ± 1.78 (range: one to 11 living children). Eighty percent of the women in the sample had used modern methods of contraception, with the oral contraceptive pill (OCP) and intrauterine devices (IUDs) being the methods most commonly used.

### 3.2. Health Service Utilisation

Table 2 shows the frequency distribution for health service utilisation by the participants. The majority of participants 80.60% had health insurance. Of those, 52.36% were covered by the private sector and 48.38% were covered by the public sector. Although most participants (87.20%) stated that they preferred that a female physician perform the Pap smear, approximately 57% reported that the presence of a male physician did not prevent them from having the test. The reported reasons for refusing to see a male physician were embarrassment, shame over exposing body parts, religious beliefs, husband's disapproval, and the ability to share feelings (including anxiety) more easily with a female physician.

Fifty-five percent of the participants reported that they had received encouragement from a healthcare provider to undergo screening, and 88.40% reported that a physician's recommendation increased the likelihood that they would be screened.

### 3.3. Cervical Cancer Screening Status

The analysis revealed that only 31.20% of the participants had actually undergone cervical cancer screening. Moreover, the majority of those women (51.28%) had had only one Pap smear.

### 3.4. Perceived Benefits, Barriers, Susceptibility, and Seriousness regarding Severity

The total mean score for perceived benefits of cancer screening was 17.80 ± 2.23, with scores ranging from five to 20. The highest mean score was recorded for the statement, “It is important for a woman to have cervical cancer screening to know if she is healthy,” and the lowest mean score was for the statement, “Cervical cancer screening can decrease the chance of a woman having an abortion.”

The total mean score for perceived barriers to cervical cancer screening was 27.10 ± 4.80, with scores ranging from 10 to 40. The highest mean score was for the statement, “My husband will not want me to do cervical cancer screening,” and the lowest mean score was for the statement, “Only women who have had babies need to do cervical cancer screening.”

The total mean score for perceived susceptibility to cervical cancer was 11.08 ± 2.16, and the scores ranged from 4 to 16. The statements, “Every woman of childbearing age is at risk of cervical cancer” and “Susceptibility to cervical cancer increases with the number of pregnancies,” both earned the highest mean score. On the contrary, the statement “Cervical cancer only happens to women who are above the age of 50 years” had the lowest mean score.

The total mean score for perceived seriousness regarding the severity of cervical cancer was 18.40 ± 2.80. The highest mean score was reported for the statement, “Cervical cancer is easily cured.” The lowest mean score was for “Cervical cancer is not as serious as other types of cancers.”.

### 3.5. Differences in Perceived Benefits, Barriers, Susceptibility, and Seriousness regarding the Severity of Cervical Cancer

Table 3 shows results of the *t*-test for differences in perceived benefits of and perceived barriers to cervical cancer screening, as well as perceived susceptibility to and perceived seriousness regarding the severity of cervical cancer among participants according to cervical cancer screening status.

There was a significant difference in perceived barriers to cervical cancer screening among women who had had a Pap smear and women who had never had one (*t* value = 4.011; *p* = 0.00). The mean for barriers was higher among women who had never had a Pap smear compared with women who had had one.

### 3.6. Predictors of Cervical Cancer Screening

Table 4 shows the results of the logistic regression analysis that was performed to identify the predictors of cervical cancer screening. The logistic regression revealed that the odds of women getting screened for cervical cancer after receiving encouragement to do so from a healthcare provider (nurse or midwife) following a Pap test were 5.24 times greater than for women who had never had a Pap test.

This result indicates that the healthcare provider's encouragement could be a factor in increasing the likelihood of cervical cancer screening. In other words, as healthcare provider encouragement increases, the odds of cervical cancer screening increase (odds ratio (OR) = 5.24, confidence interval (CI) = 95%, lower = 2.68, upper = 10.20, *p* = 0.00). Further, the OR according to years of marriage among women who had been screened for cervical cancer was 1.09 times greater than that for women who had never been screened for cervical cancer. This means that as the years being married increase, the incidence of cervical cancer screening increases (OR = 1.09, CI = 95%, lower = 1.04, upper = 1.13, *p* = 0.000).

Lastly, the OR for using a private sector healthcare provider among women who had had a Pap test was 2.20 times greater than that for women who had never had the test. In other words, women who used private sector healthcare were more likely to have a Pap test (OR = 2.20, CI = 95%, lower = 1.20, upper = 4.12, *p* = 0.012).

## 4. Discussion

### 4.1. Cervical Cancer Screening and Its Associated Factors

Findings from this study revealed that only 31% of the participants had actually been screened for cervical cancer. This finding is consistent with most of the other studies conducted in other high- to middle-income countries. A participation rate of 39.4% was reported for Qatar, where women have a Pap test once in a lifetime [15], and a rate of 23.8% was reported in a Kuwaiti study [29]. Furthermore, the incidence rate of cervical cancer screening in this study was greater than that reported by Al-Nsour et al. [13], who analysed a nationally representative sample of Jordanian women and found an incidence rate of 27.8%. This study's finding also exceeds that involving another national sample of married Jordanian women, which reported an incidence rate of cervical cancer screening of 25.7% [13].

The low incidence rate of cervical cancer screening identified in this study could be attributed to factors related to how women utilise health services. Recent data indicate that 85% of the population have health insurance. In addition, the MoH and private sector provide primary and secondary preventive healthcare services, such as screening services (DoS, 2013) [19]. However, findings of this study revealed that participating women had a low level of knowledge and awareness regarding health services, which could affect the incidence rate of cervical cancer screening. Approximately 37% of the participants reported that they had never asked if their health insurance covered the cost of a Pap smear, and 20% reported that health insurance did not cover this cost. In addition, 32% reported that they were unaware of the cost of a Pap smear, and 34% did not know if screening services were available in the health sector they utilised.

On the other hand, this study found that sociodemographic factors did not influence the screening uptake rate. This finding contrasts with those reported in previous studies in Jordan, which suggest that the low screening rate could be explained by the majority of the study sample consisting of women with low levels of education, women on a low income, and unemployed women; the result was in line with other studies [13, 16, 20].

### 4.2. Predictors of Cervical Cancer Screening among Jordanian Women

The analysis conducted for this study revealed that encouragement from a healthcare provider (nurse or midwife) to undergo cervical cancer screening was one of the predictors for screening. This finding is consistent with that reported by Amarin et al. [16], who found that most women who had at least one Pap smear (86%) did so as a result of visiting family planning clinics and receiving healthcare provider encouragement in antenatal clinics and gynaecological services. This finding highlights the crucial role of healthcare providers in increasing women's awareness of cervical cancer screening.

Results of this study indicated that the number of years of marriage was also a predictor for women's cervical cancer screening practices. The participants' mean age at marriage was 22.48 years, and the mean years of marriage was 15.5 years. With increased years of marriage, the practice of cervical cancer screening increased. It is unclear from the literature whether the number of years of marriage influences screening practices because of the limited number of studies on reproductive characteristics of women worldwide. Therefore, further studies are needed to clarify the association between years of marriage and cervical cancer screening practices.

The other predictor for screening was the use of the private sector for healthcare by women who had had a Pap test. In other words, women who used private sector healthcare providers were more likely to have this test. The private sector plays an essential role in financing and providing services. In addition, many private sector companies provide health insurance for their employees through self-insurance or pay for their employees' private health insurance. In addition, private healthcare centres spend twice as much in planning preventive measures as governmental and military healthcare sectors as discussed with WHO (2006) [30].

Findings of this study showed that the cervical cancer screening uptake was greater among women who utilised medical care provided through the private sector compared to those women who utilised medical care offered in other sectors. Specifically, women utilising private health services were 1.5 times more likely to have a Pap test than women whose medical care was provided by the MoH. These findings support those of Al-Nsour et al. [13], implying that there is a need to direct the attention of the governmental and military healthcare sectors to adopt and promote preventive approaches, such as screening services, because both sectors combined provide health insurance for more than 59% of Jordan's population.

### 4.3. Jordanian Women's Perceptions of the Benefits, Barriers, Susceptibility, and Seriousness regarding Severity

Even though the majority of the participants had adequate knowledge of the benefits of a Pap smear and a high perception of the benefits of screening, findings of this study revealed that the perceived benefits of cervical cancer screening were not predictive for the uptake of cervical cancer screening and that women's knowledge regarding the benefits did not support a positive attitude towards screening. This finding is consistent with that documented by Ibekwe [24], who indicated that the perceived benefits of cervical cancer screening do not have a positive influence on uptake. Thus, the low incidence rate of cervical cancer screening among Jordanian women could be attributed to other factors.

Most participants perceived barriers to cervical cancer screening, and there were significant differences between women who had had a Pap test and women who had never had one; the highest mean for barriers was for those who had never had a Pap smear. In other words, the women who had had a Pap test had overcome most of the perceived barriers, such as embarrassment, pain, and other obstacles. However, the majority of participants still perceived a number of obstacles that were preventing them from undergoing screening for cervical cancer. These results are consistent with those of Leyva et al. [21], who found that a significantly higher mean value of perceived barriers exists for women who have never been screened for cervical cancer compared to those who have been screened.

Results of this study also indicated that the participants highly perceived that every woman of childbearing age was at risk of cervical cancer and that susceptibility increases with an increasing number of pregnancies. Therefore, women perceived that there was a risk of developing cancer in a range of age groups not just in the older age groups and their adherence to cervical cancer screening recommendations was affected by their beliefs about the susceptibility of contracting the disease. However, these beliefs did not reinforce an uptake in screening.

Lastly, most of the participants were aware of the severity of cervical cancer and they highly perceived that the disease could be easily cured and that effective treatment was available. The majority agreed that death from cervical cancer was rare if detected early. However, despite these perceptions, the number of cervical screenings is still low, which highlights the need to explore whether other factors affect the decision to comply with the screening recommendation.

## 5. Study Limitations

This study was limited because of its cross-sectional design and the self-reporting and convenience sampling methodologies. Information bias connected with self-reporting might have influenced the findings because some women might have felt uneasy about reporting a negative response. In addition, because the participants were residents of Amman, the findings may not be generalisable to other women living in Jordan.

## 6. Implications

The study has numerous implications for nursing practices, policy makers, and researchers. Regarding nursing practices, promoting the health of women is the key to their empowerment; therefore, staff development through continuing education or in-service education programmes can be planned and implemented for nurses by using interactive methods that incorporate the use of audio-visual aids and emphasise the development of an accurate cervical cancer screening technique. On the other hand, policy makers and healthcare planners are responsible for targeting the efforts and resources in the country to increase the incidence rate of cervical cancer screening among women and finance a national programme to increase uptake. In addition, further research is needed to assess the factors associated with cervical cancer screening among Jordanian women in different settings and different governorates because rural areas, unlike Amman, may not have access to a variety of screening services.

## 7. Recommendations

Community-based screening programmes need to be designed and implemented with the cooperation of relevant national partners and institutions to change community perceptions regarding cervical cancer screening. These programmes must support men's participation and reach schools, universities, youth communities, and local community leaders. Healthcare providers, physicians, nurses, and midwives should also play their part in increasing women's awareness and knowledge about the benefits of cervical cancer screening because it has been shown that women need to be encouraged to undergo screening. Healthcare personnel also need training in performing the Pap test procedure and communicating the results of the test to women in order to allay their fears about it.

## 8. Conclusions

The results of this study revealed that the rate of cervical cancer screening among Jordanian women of all ages is low. Moreover, it was found that predictors of cervical cancer screening are healthcare providers' encouragement, years of marriage, and the use of private sector services. Furthermore, there were significant differences in perceived barriers to cervical cancer screening among women who had had a Pap smear and women who had never had one.

## Figures and Tables

**Table 1:** Sociodemographic and reproductive characteristics of participants (*n* = 500).

Characteristic	Frequency (%)
Current marital status	
Married	459 (91.80)
Divorced	19 (3.80)
Widowed	22 (4.40)
Educational level	
Less than secondary school	52 (10.40)
Secondary school	118 (23.60)
Higher education (diploma or university degree)	330 (66.00)
Current working status	
Employment	306 (61.20)
Not employed	174 (34.80)
Retired	20 (4.00)
Type of employment (*n* = 306)	
Governmental organisation	181 (59.20)
Private sector	114 (37.30)
Self-employed	11 (3.60)
Miscarriage history	
No miscarriage	284 (56.80)
Less than three abortions	179 (35.80)
More than three abortions	36 (7.20)
Previous use of a family planning method	408 (81.60)
Family planning methods	
OPC	181 (36.20)
IUD	249 (49.80)
Injectable hormone	15 (3.00)
Condom	111 (22.20)
Traditional method	115 (23.00)
Norplant	6 (1.20)
Family history of cervical cancer	17 (3.40)
History of sexually transmitted diseases	6 (1.20)

**Table 2:** Frequency distribution of health service utilisation by participants (*n* = 500).

Variable	Frequency (%)
Has health insurance	403 (80.60)
Health insurance covers the cost of Pap smear	207 (41.40)
Willing to pay the cost of Pap smear	172 (34.40)
Health services encourage women to have Pap smear	293 (58.60)
Source of regular health services	
Private sector	211 (42.20)
Public sector	195 (39.00)
Military services	139 (27.80)
Others	31 (6.20)
Has access to health services where Pap smear is available	286 (57.20)
Transportation needed	382 (76.40)
The convenience of clinic time	306 (61.20)
Preference of sex of healthcare provider doing Pap smear	
Female physician	436 (87.20)
Male physician	64 (12.80)
Healthcare provider in health facility encourages Pap smear uptake	275 (55.00)
Presence of male physician prevents having Pap smear	215 (43.00)
Physician recommendation for cervical cancer screening uptake	442 (88.40)

**Table 3:** *t*-test result for differences in perceived benefits, barriers, susceptibility, and perceived seriousness of severity among participants (*n* = 500).

Variable	Mean ± SD	df	*t* value	*p* value
Total perceived benefit				
Had Pap smear (*n* = 156)	3.60 ± .43	498	1.47	0.14
Never had Pap smear (*n* = 344)	3.54 ± .45			
Total perceived barrier				
Had Pap smear (*n* = 156)	2.58 ± .48	498	(-4.01)	^∗∗^0.0001
Never had Pap smear (*n* = 344)	2.76 ± .46			
Total perceived susceptibility to cervical cancer screening				
Had Pap smear (*n* = 156)	2.78 ± .49	498	.29	0.77
Never had Pap smear (*n* = 344)	2.76 ± .56			
Total perceived seriousness of severity of cervical cancer				
Had Pap smear (*n* = 156)	3.06 ± .46	498	.276	0.78
Never had Pap smear (*n* = 344)	3.07 ± .47			

Note: SD = standard deviation; DF = degree of freedom, *p* value < 0.0.

**Table 4:** Logistic regression analysis of predictors of cervical cancer screening for participants (*n* = 500).

Variable	*p* value	OR	95% CI	
			Lower	Upper
Healthcare provider encouragement (nurses and midwives)	.0001	5.24	2.68	10.20
Private sector	.012	2.20	1.20	4.12
Years of marriage	.0001	1.09	1.04	1.13
History of abortions				
No miscarriage	.001	.145	.044	.048
Less than three abortions	.002	.140	.041	.473
Employed	.01	.173	.045	.65

Note: OR = odds ratio; CI = confidence interval, *p* value < 0.05.

## Data Availability

Through revision of most resources that support understanding of data availability statement, all of the data supporting the finding of this study are available within the articles: research articles, documents, and books.
